# Oral Nodular Fasciitis: A Case Report in an Uncommon Location and Review of the Literature

**DOI:** 10.7759/cureus.54803

**Published:** 2024-02-24

**Authors:** Maha T Alsharif, Asma Alzahrani, Hattan Zaki, Alaa F Bukhari, Ahoud Jazzar

**Affiliations:** 1 Department of Oral Diagnostic Sciences, Faculty of Dentistry, King Abdulaziz University, Jeddah, SAU; 2 Department of Oral and Maxillofacial Diagnostic Sciences, Taibah University, Madinah, SAU

**Keywords:** spindle cell lesion, fibroblastic/myofibroblastic neoplasm, transient neoplasm, oral cavity, nodular fasciitis

## Abstract

Nodular fasciitis (NF) is a benign, self-limiting condition that is often misdiagnosed due to its resemblance to other lesions. Although NF is common, its occurrence in the oral cavity is rare and particularly challenging for both clinicians and pathologists. To date, no case has been reported in the retromolar area of the oral cavity.* *A 49-year-old male patient presented with a painless, rapidly growing, firm nodule in the right retromolar area. Histopathological examination revealed spindle cell proliferation with characteristics of NF and immunohistochemical analysis confirmed the diagnosis. The lesion was treated by conservative surgical excision, without recurrence at a one-year follow-up. In the current case, 54 cases of oral nodular fasciitis (ONF) have been documented. The majority of ONF-affected individuals are in their 40s, with a 1:1 male-to-female ratio. The buccal mucosa was the most commonly involved site followed by the tongue and labial mucosa. Histopathologically, the most prominent features were the proliferation of uniform spindle-shaped cells within a myxomatous and/or fibrotic background. A positive smooth muscle actin (SMA) stain was a consistent finding. Complete local excision remains the preferred treatment method, and no recurrences have been reported. This report underscores the importance of considering NF in the differential diagnosis of oral spindle cell lesions and emphasizes the need for a comprehensive evaluation to guide appropriate management.

## Introduction

Nodular fasciitis (NF) is a benign self-limiting pathological entity of a fibroblastic/myofibroblastic origin that typically affects young adults [1,2]. NF poses diagnostic challenges, both clinically and microscopically, with more than half of the cases misinterpreted as malignancy [1-3]. NF was first named as pseudosarcomatous fasciitis by Konwaler et al. in 1955 due to the clinical features of rapid and infiltrative growth and histopathological findings of spindle cells with frequent mitotic activity [4]. Since then, other synonyms have been used in the literature, including nodular fibrositis, subcutaneous fibromatosis, proliferative fasciitis, subcutaneous pseudosarcomatous fibromatosis, and infiltrative fasciitis [5]. Notably, the clinical course of NF is entirely benign and is often managed by conservative surgical excision with a minimal local recurrence rate [3,6,7]. The cause of NF has long been described as uncertain, and researchers have suggested that NF may be triggered by trauma, local infections, or inflammation, including biopsy [2,7,8]. It also was hypnotized that NF represents a reactive process due to the characteristics of self-limiting and spontaneous regression. However, due to emerging evidence for the identification of novel MYH9-USP6 fusion among the majority of NF cases, NF is now recognized as a “transient” neoplasm [1]. Clinically, NF is often present as a rapidly growing firm nodule found in the facia and subcutis of the upper extremities and trunk. The head and neck area account for nearly 10-20% of the cases, with exceedingly rare involvement of intra-oral locations [4,7]. Among the cases of oral nodular fasciitis (ONF) published in the literature, buccal mucosa, labial mucosa, and tongue are the most frequently affected sites [5,9,10]. Although NF is rare in this location, its deceptive nature warrants clinicians and pathologists to include NF in the differential diagnosis of spindle cell lesions in the oral cavity and to cautiously distinguish it from malignancy to avoid unnecessary overtreatment [11]. Herein, we describe a unique case of ONF involving the retromolar area in an adult patient with a review of the literature regarding the clinical and histopathologic characteristics of the oral cases.

## Case presentation

**Figure 1:**
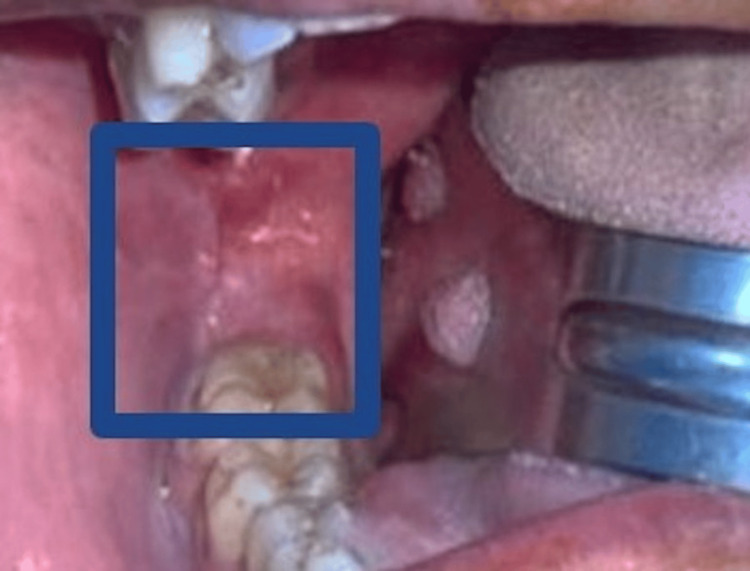
Intraoral photograph showing submucosal mass noted in the right retromolar area, behind the lower right second molar.

A healthy 49-year-old Caucasian male presented to the oral and maxillofacial surgery department for consultation on painless swelling in the right retromolar area. The patient reported that the lesion first appeared five weeks prior to the presentation. He denied any pain or discomfort linked to the lesion. He also denied any previous history of trauma or other diseases in the area. His medical and family history were non-contributory with no history of using tobacco. Upon intraoral examination, a submucosal mass was noted in the right retromolar area behind the lower right third molar (Figure 1). The nodule was firm in consistency upon palpation, with a slightly ulcerated surface and no signs of acute inflammation in the area. The nodule’s largest diameter was approximately 0.5 cm. No palpable lymph nodes were detected. The patient had good oral hygiene and a normal mouth opening. An examination of the cranial nerves was within normal limits. The clinical impression was a reactive or benign lesion. An incisional biopsy was performed. The microscopic diagnosis was established as a spindle cell lesion without characterization, and further investigations were recommended.

Radiographic investigation

**Figure 2:**
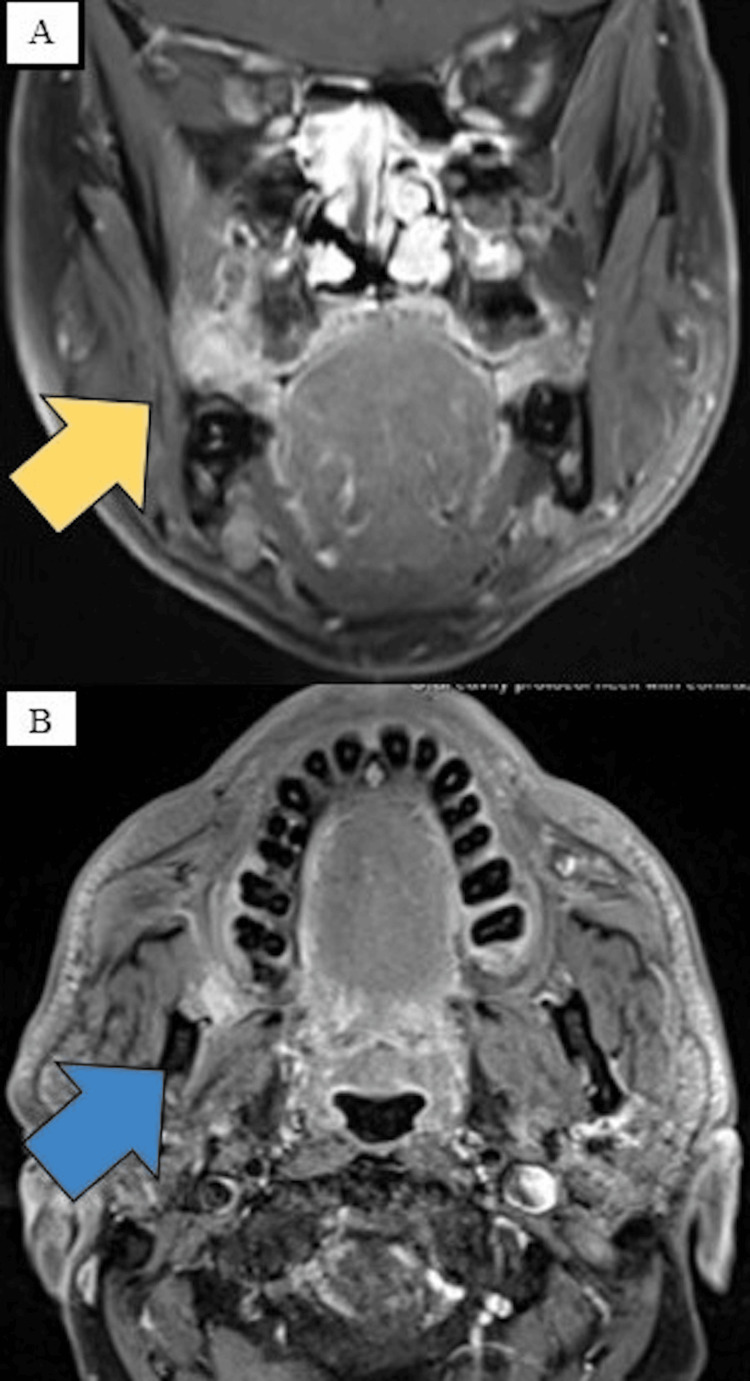
(A) Contrast-enhanced MRI imaging shows a soft tissue lesion in the right retromolar extending superiorly to the level of the maxillary alveolus (yellow arrow). No destruction of the underlying bone was observed. The lesion measurement was about 1.5 x 0.9 x 1.6 cm. (B) The soft tissue enhancement reaches the anterior inferior aspect of the lateral pterygoid muscle (blue arrow).

The patient returned two weeks after the incisional biopsy complaining that the lesion had doubled in size. Contrast-enhanced MRI (Figures 2A-2B) revealed a right retromolar soft tissue lesion extending superiorly to the level of the maxillary alveolus. No destruction of the underlying bone was observed. The lesion measurement was about 1.5 x 0.9 x 1.6 cm. The patient was scheduled for surgery to excise the remaining lesion and rule out the possibility of malignancy. Conservative surgical excision was done under general anesthesia and the specimen was sent for histopathological examination of the surgical specimen.

Histopathological examination and diagnosis

**Figure 3:**
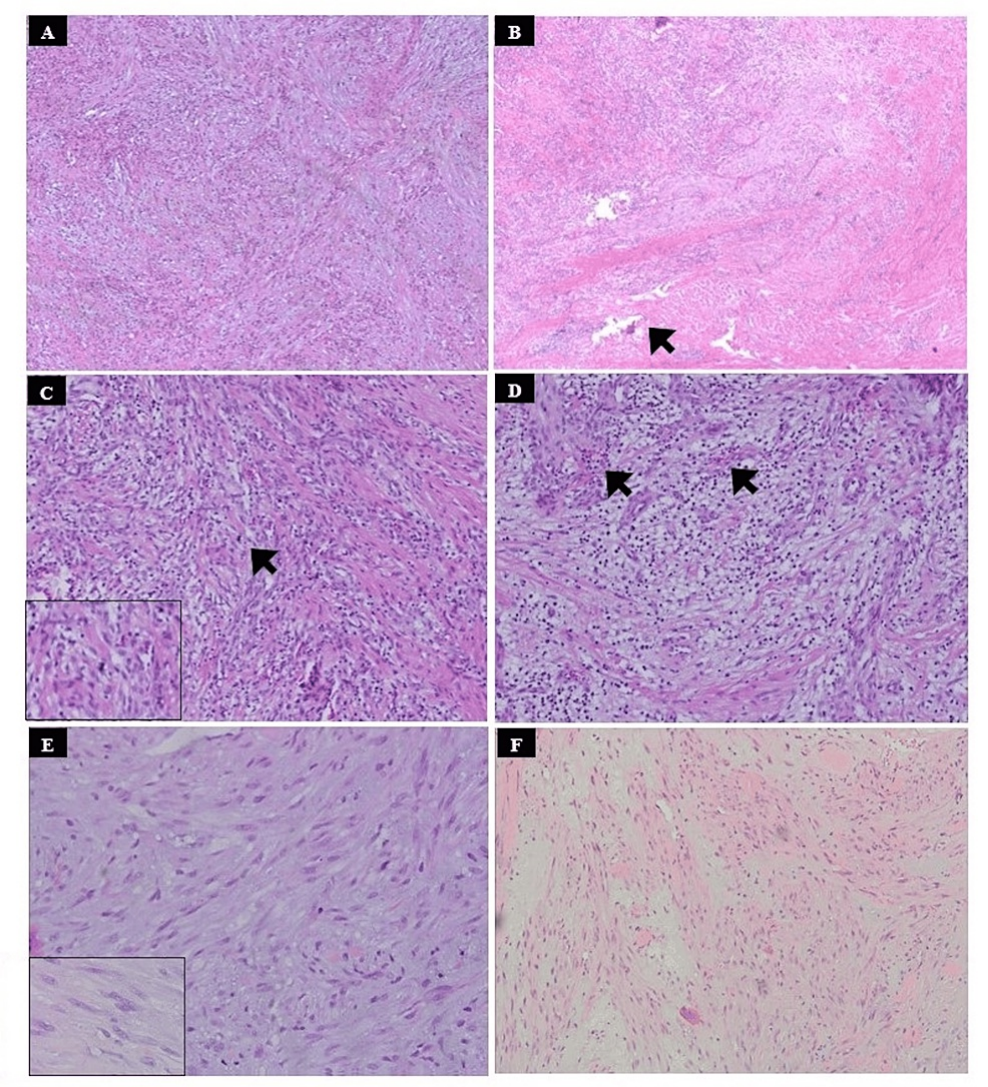
Photomicrographs (hematoxylin and eosin, original magnification Χ100); (A) Tumour cells arranged in short and long fascicular architecture. (B) Showing uniform proliferation of plumped spindle cells, with vesicular nuclei, poorly defined borders and eosinophilic cytoplasm. (C) Illustrating the process of mitosis (arrow) (D) Demonstrating area of the loosely textured arrangement of fibroblasts-like cells in the myxomatous background; (E) Showing inflammatory cells and extravasated erythrocytes. (F) Tumour cells are arranged in the feathery pattern entrapped of adjacent skeletal muscle fibres between the spindle.

Microscopic examination of hematoxylin and eosin stained sections (Figures 3A-3F) showed uniform proliferation of plumped spindle cells, with vesicular nuclei, poorly defined borders and eosinophilic cytoplasm arranged in short and long fascicular architecture. The stroma showed both fibrotic and myxoid areas with extravasated erythrocytes and intense chronic inflammatory cells infiltrate, mainly of lymphocytes, in the background. Rare mitotic figures were observed. Tumor cells were found in close relation to the adjacent skeletal muscle fibers. Based on the clinical presentation of rapidly growing and locally infiltrative mass in the retromolar area with the histopathological features of spindle cell proliferation, the differential diagnosis of NF, leiomyosarcoma, low-grade myofibroblastic sarcoma, and spindle cell carcinoma was considered.

**Figure 4:**
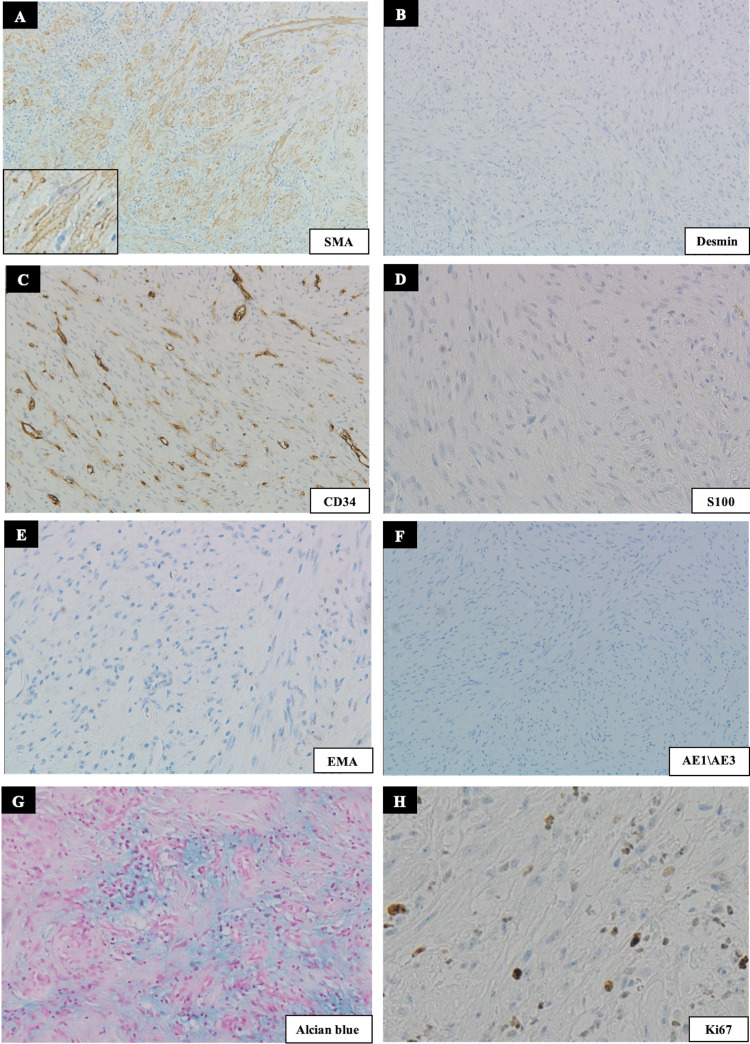
Photomicrographs showing (A) Positive staining for SMA with a tram-like pattern (inset); (B) Tumour cells show lack of staining for desmin; (C) CD34; (D) S-100; (E) EMA, and (F) pancytokeratin; (G) The background is positive for Alcian blue staining highlighting the mucopolysaccharide-rich matrix. (H) The Ki67 shows nuclear staining with a proliferation index of approximately 5-10%.

To reach a diagnostic conclusion, the following immunohistochemical (IHC) panel was performed (Figures 4A-4H): smooth muscle actin (SMA), S100, epithelial membrane antigen (EMA), CD34, CD99, pancytokeratin (AE1/AE3), desmin, p63, and S-100, and Ki-67. Alcian blue chemical stain was also performed. IHC stains were all negative for tumor cells except for SMA, which was diffusely positive with a tram-line pattern supporting the myofibroblastic origin for the tumor cells. The Ki67 showed nuclear staining with a proliferation index of approximately 5-10%. The background was positive for Alcian blue staining, highlighting the mucopolysaccharide-rich matrix. Based on the clinical features of rapid growth, histomorphological features and positive SMA and Alcian blue stains, the definitive diagnosis was consistent with NF. At the one-year follow-up, the patient reported no pain or discomfort in the area and showed no signs of recurrence. The patient continues to be monitored.

## Discussion

NF is a well-established soft tissue lesion, yet it remains one of the most commonly misdiagnosed neoplasms. Although the head and neck area is one of the common locations of NF onset, its prevalence in the oral cavity is rare, with nearly 50 cases documented in the literature [12]. NF of the oral cavity poses a diagnostic challenge due to its rarity in this region on the one hand and its non-characteristic histopathological features on the other hand [13]. This case report is unique because of its occurrence in an uncommon location.

NF was long believed to represent a reactive or inflammatory condition that is triggered by local injury or infection rather than a true neoplasm. Historically, trauma was linked to NF as a potential etiology. However, in a large series study, only five of 114 patients reported a history of trauma. Also, the recent discovery of a novel gene mutation detected among NF cases has led to the reclassification of this entity as a neoplasm of fibroblastic/myofibroblastic lineage. Erickson-Johnson et al. were the first to report high expression of recurrent rearrangement within the USP6 locus on ch17p13 with MYH9 as the most commonly identified fusion partner [11]. Moreover, the tendency toward self-limiting and spontaneous regression has proposed a novel classification of this entity as a “transient neoplasia” [1]. The driving mechanism by which the USP6 oncogenic fusion event produces the unique clinical behavior of NF is still poorly understood [14]. The USP6 gene encodes a ubiquitin protease enzyme that regulates different cellular processes, and it is also well-known to be overexpressed in other soft tissue and bone lesions, which are referred to as USP6-associated neoplasms (UNA). The UNA family includes, alongside NF, aneurysmal bone cyst, myositis ossificans and fibro-osseous pseudotumor of digits and fibroma of tendon sheath, all of which are indolent in nature and share similar biological and histopathological spectrum [10]. For cases in which the morphological features and IHC stains are not completely conclusive, molecular analysis of the USP6 rearrangement by fluorescent situ hybridization (FISH) or next-generation sequencing (NGS) has proven to be a sensitive and specific diagnostic tool [11].

For the present paper, a search of the English-language literature regarding ONF yielded 42 studies with a total of 53 cases. Table 1 summarizes the clinical features of ONF reported in the literature including our case. NF typically occurs in young adults between the ages of 20 and 40 years, with slight female predilection [2]. Based on the pooled data from the 53 ONF cases, the age of patients ranged from infancy (four months old) to elderly (78 years old), with a mean age of 39 years and a 1:1 male-to-female ratio. In our case, the lesion occurred in a 49-year-old male, who is considered slightly older than previously described ONF patients. This finding contracts border reviews of NF, which reported that the majority of orofacial lesions occurred during childhood and infancy [9].

**Table 1:** Demographic data, clinical features, treatment and follow-up information of 54 cases of ONF BM: buccal mucosa, F: female, FOM: floor of the mouth, M: male, Mo: month NA: not applicable, N.E.D.: no evidence of disease, y: year

Case	Authors	Age	gender	Location	Size (cm)	Duration	Sign and symptoms	Clinical presentation	Growth rate	Treatment	Follow up
1	Smith [15]	42 y	M	BM	NA	9 mo	Tenderness	Mobile mass	Rapid	Excision	NA
2	Abulafia et al. [16]	65 y	M	Lower lip	NA	NA	Asymptomatic	Hard, elastic submucosal mass	NA	Excision	NA
3	Lummerman et al. [17]	31 y	F	BM	2	3 day	Tenderness	Mobile, firm submucosal mass	Rapid	Excision	2.5 y/N.E.D
4	Solomon et al. [18]	47 y	M	BM	3	3 mo	Asymptomatic	Palpable submucosal mass	Slow	Excision	1 mo/N.E.D
5	Larsson and Svartz [19]	25 y	F	BM	1	3 day	Painful	Mobile submucosal mass	Rapid	Excision	4 y/N.E.D
6	Werning [20]	NA	NA	BM	NA	NA	NA	NA	NA	NA	NA
7	Werning [20]	NA	NA	BM	NA	NA	NA	NA	NA	NA	NA
8	Werning [20]	NA	NA	BM	NA	NA	NA	NA	NA	NA	NA
9	Werning [20]	NA	NA	Tongue	NA	NA	NA	NA	NA	NA	NA
10	Sato et al. [21]	31 y	M	Gingiva	4	2 mo	Painful	Ulcerative mass, causing bone erosion	NA	Excision	1 y/N.E.D
11	Takagi and Ishikawa [22]	34 y	M	Tongue	1.5	1 mo	Asymptomatic	Ulcerated submucosal mass	NA	Excision	NA
12	Takagi and Ishikawa [22]	46 y	F	Palate	1	NA	Asymptomatic	Submucosal mass	NA	Excision	NA
13	Kawana et al. [23]	42 y	M	Upper lip	2	1 mo	Tenderness	Erythematous mobile mass	Rapid growth after the incisional biopsy	Excision	NA
14	Freedman and Lumerman [24]	19 y	F	Buccal vestibule	2.5	3 mo	NA	Ulcerated firm mass	NA	Excision	Several mo/N.E.D
15	Freedman and Lumerman [24]	53 y	M	BM	2	2 mo	NA	Firm mass	NA	Excision	Several mo/N.E.D
16	Kahn et al. [25]	20 y	F	Lower lip	1.5	3 mo	Asymptomatic	Firm submucosal mass	Slow	Excision	3 mo/N.E.D
17	Davies et al. [26]	15 y	M	Chin and labial sulcus	1	1 mo	Painful	Firm submucosal mass	Rapid growth after the incisional biopsy	Excision	6 mo/N.E.D
18	Bodner and Dayan [27]	20 y	M	Upper lip	2	1 mo	Asymptomatic	Mobile, firm submucosal mass	Slow	Excision	1 y/N.E.D
19	Shlomi et al. [28]	30 y	F	BM	2	2 mo	Tenderness	Ulcerated, indurated firm mass	Rapid	Excision	6 mo/N.E.D
20	Badia et al. [29]	76 y	F	BM	1.8	6 mo	NA	Firm mass	NA	Excision	1.5 y/N.E.D
21	Haddad et al. [30]	9 y	F	Upper lip	1	3 weeks	Asymptomatic	Mobile submucosal mass	Rapid	Excision	NA
22	Alkan et al. [31]	35 y	F	BM	2	4 mo	Painful	Mobile, indurated submucosal mass	NA	Excision	15 mo/N.E.D
23	Martinez-Blanco et al. [7]	73 y	M	FOM	2	1 mo	Asymptomatic	Firm mass	Rapid	Excision	4 y/N.E.D
24	Martinez-Blanco et al. [7]	53 y	M	Tongue	2.5	3 weeks	Asymptomatic	Hard mass	Rapid	Excision	3 mo/N.E.D
25	Nair et al. [32]	37 y	F	BM	1.5	2 mo	Asymptomatic	Mobile, soft submucosal mass	Slow	Excision	18 mo/N.E.D
26	Dayan et al. [9]	43 y	F	BM	2	3 weeks	Asymptomatic	Firm submucosal mass	NA	Excision	1 y/N.E.D
27	Dayan et al. [9]	42 y	F	BM	1	2 y	Tenderness	Rubbery mass	NA	Excision	2 y/N.E.D
28	Dayan et al. [9]	50 y	F	BM	2	2 mo	Asymptomatic	Ulcerated mass with indurated margin	NA	Excision	4 y/N.E.D
29	Dayan et al. [9]	38 y	M	BM	NA	NA	Asymptomatic	Mass	Rapid	Excision	5 y/N.E.D
30	Dayan et al. [9]	37 y	F	FOM	4	NA	Asymptomatic	Mass	NM	Excision	7 y/N.E.D
31	Han et al. [33]	47 y	F	Gingiva	3.5	1 mo	Painful	NA	NA	Excision	4 y/N.E.D
32	Han et al. [33]	48 y	F	BM	2	4 weeks	Asymptomatic	Firm submucosal mass	Rapid	Excision	9 y/N.E.D
33	Leventis et al. [34]	50 y	F	BM	1٫5	8 mo	Asymptomatic	Soft to firm mass	NA	Excision	1 y/N.E.D
34	Naidu and Lerman [35]	28 y	M	Buccal vestibule	2٫5	3 weeks	Asymptomatic	Ulcerated soft mass	Rapid	Excision	1 y/N.E.D
35	Reiser et al. [36]	58 y	F	BM	1.7	NA	Asymptomatic	Mobile mass	Rapid	Excision	1 y/N.E.D
36	Subramaniam et al. [37]	9 y	M	BM	3	1 mo	Painful	Firm mobile mass	Rapid	Excision	NA
37	Chi et al. [38]	20 y	F	Upper lip	0.6	3 weeks	Asymptomatic	Firm submucosal mass	Rapid	Excision	NA
38	Imai et al. [39]	36 y	F	Gingiva	4	1 mo	Discomfort	Soft to firm mass	NA	Excision	4 y/N.E.D
39	Seo et al. [40]	26 y	M	Lower lip	1	1 mo	Asymptomatic	Firm submucosal mass	Rapid	excision	2 mo/N.E.D
40	de Carli et al. [13]	32 y	M	BM	2	45 day	Asymptomatic	Firm erythematous mass	Rapid	Excision	3 mo/N.E.D
41	Lima et al. [41]	18 y	F	Gingiva	1٫3	3 mo	Painful	Ulcerated, firm mass	NA	Excision	3 mo/N.E.D
42	Celentano et al. [42]	67 y	M	Tongue	2	3 mo	Asymptomatic	Ulcerated, firm submucosal mass	Rapid	Excision	5 mo/N.E.D
43	Lloyd et al. [43]	51 y	F	Lower lip	1.5	3 weeks	Asymptomatic	Firm and fixed mass	Rapid	Excision	7 mo/N.E.D
44	Kuklani et al. [44]	25 y	F	FOM	1	NA	Asymptomatic	Mobile submucosal mass	Rapid	Excision	2 y/N.E.D
45	Kuklani et al. [44]	26 y	M	Tongue	1	2 weeks	Asymptomatic	Ulcerated, firm mass	Rapid	Excision	Recurrent after 16 days, N.E.D after 1.5 y
46	Souza et al. [45]	17 y	F	Buccal vestibule	NA	7 weeks	Asymptomatic	Rubbery mass	NA	Excision	8 mo/N.E.D
47	Chemmanam [46]	78 y	M	Tongue	2	NA	Discomfort	Indurated mass	NA	Excision	18 mo/N.E.D
48	Shupak et al. [47]	4 mo	M	Tongue	2	1 week	Discomfort	Firm mass	Rapid	Excision	2 mo/N.E.D
49	Zhurakivska et al. [48]	42 y	M	BM	1٫5	NA	Asymptomatic	Ulcerated mass	NM	Excision	3 y/N.E.D
50	Rathna et al. [49]	30 y	M	Buccal vestibule	3	3 mo	Tenderness	Lobulated, firm mass	Slow	Excision	8 mo/N.E.D
51	Khan et al. [25]	62 y	M	Buccal and lingual vestibules	5	3 mo	Asymptomatic	Firm mass	NA	Excision	NA
52	Yoshizawa et al. [50]	72 y	M	BM	5	2 mo	NA	Soft to firm mass	Rapid	Excision	2 y/N.E.D
53	Koubik et al. [51]	27 y	F	Tongue	1٫3	2 mo	Asymptomatic	Firm mass	Rapid	Excision	18 mo/N.E.D
54	Our case	49 y	M	Retromolar area	1.5	5 weeks	Asymptomatic	Ulcerated firm mass	Rapid growth after the incisional biopsy	Excision	1 y/N.E.D

NF could arise in any anatomical location where the upper extremity is the most common location (34%), followed by the head and neck region (24%), trunk (21%), and lower extremity (14%) [52]. With respect to the oral cavity, the most commonly reported intraoral sites were the buccal mucosa (n = 23), followed by the tongue and labial mucosa (n = 8 each). There is no reported variation in terms of clinical features, rate of growth, or aggressiveness between ONF and other anatomic locations [41].

Clinically, NF is usually described as a well-circumscribed single mass ranging from 1-3 cm in diameter and usually not exceeding 5 cm. Oral cavity lesions are mostly described as exophytic lesions or submucosal nodular masses that are firm in consistency. Some of the lesions are associated with surface ulceration and/or bone erosion. Concurrent multiple nodules in the same or at different anatomical sites have been rarely reported but none was documented in the oral cavity [53]. The clinical appearance of NF is not entirely specific but has the potential for self-limiting is characteristic. The tentative clinical diagnosis of intraoral lesions varies from benign mesenchymal tumours, such as fibroma, irritational fibroma, and peripheral giant cell granuloma, to malignant lesions such as mesenchymal sarcoma, hematological malignancy, and spindle cell carcinoma. NF is subdivided into subcutaneous (most common), intramuscular, and fascial based on its relationship with the fascia [34]. Other rare subtypes that have also been described in the oral cavity are intravascular fasciitis, ossifying fasciitis, and proliferative fasciitis [2,44].

Typically, NF grows rapidly over a two to three-week period in most cases. A mean duration of 2.4 months was reported for 42 patients with ONF. The growth rate ranged from sudden and rapid growth of three days duration to gradual and slow growth over three years, with three cases, including our case, demonstrating rapid growth after incisional biopsy. As in our patient asymptomatic or pain-free lesions were described in the majority of the patients (n = 30). Signs and symptoms, such as pain, discomfort, tenderness, and difficulty during eating, are not prominent features, occurring in only 16 cases. In these instances, pain could be attributed to the pressure from the lesion on an adjacent nerve [44].

Table 2 summarizes the histopathological features of the previously reported ONF cases. On histologic examination, the typical features of NF, including ONF, are composed of well-circumscribed yet non-encapsulated masses with proliferation of fibroblastic/myofibroblastic cells that are arranged in short fascicles or haphazard bundles. In highly cellular areas, the cells may be arranged in a storiform pattern, S-shaped or C-shaped fascicles. The cells usually appear plumped and spindle-shaped, with vesicular nuclei and prominent nucleoli and eosinophilic cytoplasm. However, nuclear hyperchromasia and prominent pleomorphism are not typically observed, as these features are generally absent [12,52]. In the majority of ONF cases, the presence of mitotic figures ranges from absent or few to abundant; this differs from what is described for NF lesions located in other locations, which typically show numerous mitotic figures. Careful evaluation to identify atypical or brisk mitoses should be pursued, as such a finding is not consistent with a diagnosis of NF. The cells at the lesion border are typically found infiltrative between surrounding tissues. Other findings, such as scattered extravasated erythrocytes or microhemorrhage (n=17), lymphocytic infiltration (n = 32), and osteoclast-like giant cells (n = 8), were also reported in ONF cases. The stroma may demonstrate high cellularity, hyalinization, and/or myxoid (mucopolysaccharide-rich) areas [9,52]. The presence of abundant mucopolysaccharide stroma is believed to contribute to the distinctive ‘tissue culture-like’ or ‘feathery’ appearance [9]. The challenges in histopathologic diagnosis of NF arise from its non-specific features; however, the loose feathery pattern with mucopolysaccharide-rich intervening matrix is an important diagnostic criterion for NF [1]. The histological characteristics of our case are similar to those previously documented. The histopathological differential diagnosis includes a diversity of benign and malignant spindle cell lesions. On the one hand, the benign entities may include fibrous histiocytoma, myofibroma, peripheral nerve sheath tumors, and smooth muscle tumors. While on the other hand, fibrosarcoma, spindle cell carcinoma, low-grade myofibroblastic sarcoma, and other sarcomas are malignant neoplasms with spindle cell proliferation that must be distinguished from NF. To differentiate NF from previously mentioned malignancies, careful evaluation to identify atypical mitoses, prominent pleomorphism, necrosis, long sweeping bundles, and herringbone patterns, should be pursued, as such findings are not consistent with a diagnosis of NF [33].

**Table 2:** The reported histopathologic characteristics and immunohistochemical findings of ONF cases ONF: oral nodular fasciitis

Lesion border		Pattern		Mitosis		Stroma			Other				Subtype		Stain							
Well circumscribed	Peripheral extension	Bundles	Discohesive	High	Low/absent	Fibrotic	Myxoid/feathery/culture	Both	Extravasated RBCS	Inflammatory cells	Giant cell	Calcification	Classic	Intravascular	Vimentin	Muscle-specific actin (HHF-35)	Smooth muscle actin (SMA)	Desmin	Pancyto-keratin	S-100	CD34	Alcian blue
21	15	26	15	13	15	7	17	15	18	33	8	1	45	9	17/17	8/13	21/24	0/19	0/19	0/23	0/11	4/4

IHC analysis is helpful in reaching the diagnosis and excluding other differential diagnoses. The tumor cells typically express vimentin, SMA, and muscle-specific actin in a tram-track pattern that highlights the myofibroblastic differentiation of the spindle cells. Focal desmin expression is occasionally found. The stroma can be positive for Alcian blue highlighting mucopolysaccharide-rich background. The tumor cells should be consistently negative for S-100 protein, cytokeratin markers, and CD34 [9,52]. The IHC stains were reported in some studies of ONF (Table 2).

All cases of ONF were treated by complete surgical excision with an entirely benign course. Only one case showed spontaneous regression after the incisional biopsy. Follow-up information was available for 39 cases. Among all cases, only a single case reported a recurrence 16 days after incomplete excision. However, no recurrence was noted during the one-year and five-month follow-up after the complete re-excision. Therefore, in cases of recurrent NF, incomplete resection or malignancy should be suspected [53]. In the present case, we performed a complete surgical excision with margins and primary closure using the buccal fat pad while ensuring the preservation of facial nerve integrity. After one year of follow-up, no recurrence was detected.

## Conclusions

In conclusion, NF is a benign lesion that poses a diagnostic challenge. Despite its rarity in the retromolar area of the oral cavity, clinicians and pathologists must have an adequate understanding of NF and include it in the differential diagnosis of spindle cell lesions to avoid misdiagnosis and unnecessary overtreatment. Moreover, the correlation of clinical features with histopathologic findings and the IHC profile is extremely vital for the diagnosis of NF.
